# Muscle-Related Plectinopathies

**DOI:** 10.3390/cells10092480

**Published:** 2021-09-19

**Authors:** Michaela M. Zrelski, Monika Kustermann, Lilli Winter

**Affiliations:** Center for Anatomy and Cell Biology, Neuromuscular Research Department, Medical University of Vienna, 1090 Vienna, Austria; michaela.zrelski@meduniwien.ac.at (M.M.Z.); monika.kustermann@meduniwien.ac.at (M.K.)

**Keywords:** plectin, muscular dystrophy, myopathology, desmin, intermediate filaments, sarcomere structure

## Abstract

Plectin is a giant cytoskeletal crosslinker and intermediate filament stabilizing protein. Mutations in the human plectin gene (*PLEC*) cause several rare diseases that are grouped under the term plectinopathies. The most common disorder is autosomal recessive disease epidermolysis bullosa simplex with muscular dystrophy (EBS-MD), which is characterized by skin blistering and progressive muscle weakness. Besides EBS-MD, *PLEC* mutations lead to EBS with nail dystrophy, EBS-MD with a myasthenic syndrome, EBS with pyloric atresia, limb-girdle muscular dystrophy type R17, or EBS-Ogna. In this review, we focus on the clinical and pathological manifestations caused by *PLEC* mutations on skeletal and cardiac muscle. Skeletal muscle biopsies from EBS-MD patients and plectin-deficient mice revealed severe dystrophic features with variation in fiber size, degenerative myofibrillar changes, mitochondrial alterations, and pathological desmin-positive protein aggregates. Ultrastructurally, *PLEC* mutations lead to a disorganization of myofibrils and sarcomeres, Z- and I-band alterations, autophagic vacuoles and cytoplasmic bodies, and misplaced and degenerating mitochondria. We also summarize a variety of genetically manipulated mouse and cell models, which are either plectin-deficient or that specifically lack a skeletal muscle-expressed plectin isoform. These models are powerful tools to study functional and molecular consequences of *PLEC* defects and their downstream effects on the skeletal muscle organization.

## 1. Introduction

**Figure 1:**
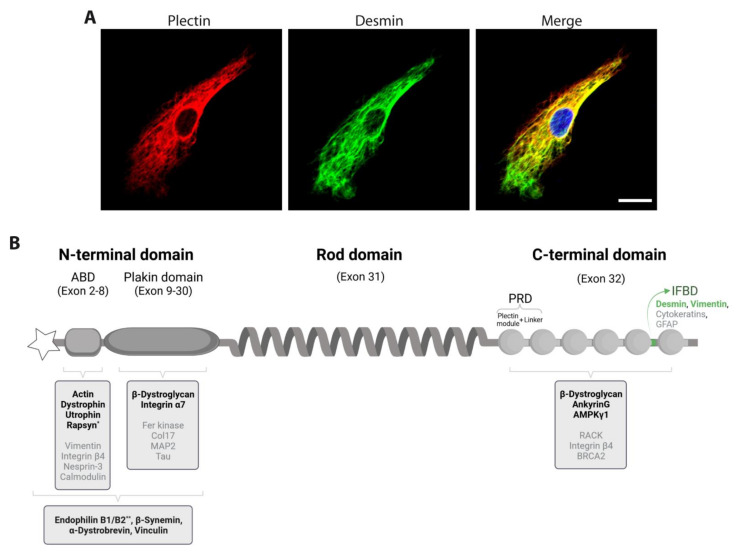
Subcellular localization of plectin in a myoblast and schematic representation of plectin and its binding partners, with a special focus on direct interaction partners identified in myoblasts and/or skeletal muscle. (**A**) Immunofluorescence microscopy of a human primary myoblast using antibodies to plectin (in red) and desmin (in green). The nucleus was visualized using DAPI (blue in the merged image). Note the co-localization of plectin and desmin. Scale bar: 15 µm. (**B**) Schematic domain map of the protein. The tripartite structure of plectin comprises a central rod domain (encoded by exon 31) flanked by N- and C-terminal domains. The N terminus harbors differentially spliced first exons (star), an actin-binding domain (ABD, exons 2–8), and a plakin domain (exons 9–30), while the C terminus comprises six plectin repeat domains (PRD), each containing a conserved core (plectin module) and a linker region. An intermediate filament binding domain (IFBD) is located between modules 5 and 6 (green). Binding partners are indicated below the scheme; binding partners which were experimentally found in myoblasts/skeletal muscle are highlighted in bold. * Interaction was shown for isoform P1f. ** Interaction was shown for isoform P1.

Cardiac and skeletal striated muscles are elaborately organized machines designated for contraction. Sarcomeres, the smallest functional units of muscle contraction, comprise precisely organized filament systems including thin (actin) and thick (myosin) filaments, titin, and nebulin [1] and build up the myofibrillar apparatus. Desmin intermediate filaments (IFs), which are structurally organized by plectin in myoblasts (Figure 1A) and muscle fibers, constitute the principal component of the extrasarcomeric cytoskeleton. Plectin, a giant (>500 kDa) multi-modular cytolinker of the plakin protein family [2], may be considered as the universal cross-linking element of the cytoskeleton. Possessing binding sites for all types of IF subunit proteins, it networks and anchors them to sites of strategic importance for the organization and performance of cells, such as transmembrane junctional complexes, the nuclear envelope, and cytoplasmic organelles [3]. In addition, plectin harbors a functional actin-binding domain (ABD); binds to microtubule-associated proteins (MAPs); and interacts with transmembrane receptors, proteins of the subplasma membrane protein skeleton, components of the nuclear envelope, and several kinases with known roles in the migration, proliferation, and energy metabolism of cells (Figure 1B) [4,5]. Plectin’s functional versatility is not only due to its multi-domain structure enabling a broad range of interactions, but it is also due to an unusual transcript diversity that is largely based on at least nine different, relatively short N-terminal sequences. Encoded by alternatively spliced first exons [6], individual plectin isoforms are differentially targeted to distinct cellular locations where they function as universal IF-docking sites, thus enabling them to fulfill distinct functions in different cell types and tissues [4,5]. In muscle tissue, the four most prominently isoforms expressed (plectin isoform 1d (P1d), P1f, P1b, and P1) are crucial for myofiber integrity by anchoring desmin IFs to Z-disks, costameres, mitochondria, and the nuclear/sarcoplasmic reticulum membrane system, respectively [7,8,9]. Thus, plectin acts as a multi-functional linker and signaling scaffold, centrally orchestrating the structural and functional organization of filamentous cytoskeletal networks and thereby substantially contributing to the fundamental biomechanical properties of stress-bearing tissues such as muscle.

## 2. Human Plectinopathies

The proposed concept that plectin contributes to the stability and coherence of cells was confirmed by showing that mutations in the human plectin gene (*PLEC*, NM_000445) on chromosome 8q24 cause a variety of human disorders referred to as “plectinopathies”. Plectinopathies belong to the group of rare diseases with an incidence of less than 5 affected individuals for every 10,000 people. As of today, almost 100 disease-causing *PLEC* gene alterations comprising missense, frame-shift, and splice site mutations as well as small in-frame deletions have been reported. Most *PLEC* mutations cause epidermolysis bullosa simplex with muscular dystrophy (EBS-MD, MIM #226670), an autosomal recessive skin blistering disorder associated with progressive muscle weakness [4]. A plethora of additional symptoms has been described for EBS-MD patients in recent years, including cardiac pathology, nail deformation, tooth decay, erosive lesions on the oral or laryngeal mucosa, hoarseness, respiratory complications during infant life, and urethral strictures. In addition to EBS-MD, *PLEC* mutations have been shown to lead to EBS-MD with a myasthenic syndrome (EBS-MD-MyS) or EBS with pyloric atresia (EBS-PA, MIM #612138) [4]. Autosomal dominant *PLEC* mutations (c.5998C > T, p.R2000W; c.8668A > T, p.T2890S; and c.10579C > T, p.R3527C) cause EBS-Ogna (MIM #131950), where the patients suffer from generalized skin blistering or fragile skin without showing any muscular symptoms [10,11,12]. Recently, the first mutations in alternative first exons have been described. A homozygous 9 bp deletion (c.1_9del1f) containing the initiation codon of exon 1f (and therefore resulting in the loss of isoform P1f) was identified in several patients suffering from limb-girdle muscular dystrophy (LGMDR17, previously denoted as LGMD2Q [13], MIM #613723); however, these patients did not show any overt signs of an epidermolytic skin disease [14,15]. Since then, another exon 1f-specific mutation (c.58G > T, p.E20X) has been reported, where three siblings suffered from MD and respiratory problems but who did not present with any skin involvement [16]. Likewise, a homozygous nonsense mutation in exon 1a (c.46C > T), leading to a premature termination codon p.R16X and therefore to the disruption of P1a, an isoform that is hardly expressed in muscle, resulted in a skin-only EBS phenotype without muscle involvement (EBS with nail dystrophy, EBSND, MIM #616487) [17]. Taken together, plectinopathies emerge as complex multi-systemic disorders, primarily affecting tissues exposed to great mechanical stress such as skin and muscle but including more and more additional symptoms and disease manifestations.

## 3. Clinical Phenotypes and Muscle-Related Disease Manifestations of Human Plectinopathies

### 3.1. EBS-MD, the Most Common Muscle-Related Plectinopathy 

While EBS-MD patients usually suffer from skin blistering early in life, muscle-specific symptoms can occur between early infancy up to the fourth decade of life, with first signs such as gross developmental motor delays (e.g., delayed independent walking), fatigability, muscle weakness, predominantly affecting proximal upper and lower muscle groups, and ptosis. Disease progression is relatively slow; most affected individuals noted the first signs of distal or proximal muscle weakness in the second decade of life. Loss of ambulation was reported for EBS-MD patients between 14 and 47 years of age. Out of 53 EBS-MD cases with genetically determined *PLEC* mutations (see Table 1), muscular symptoms have been described for 37 patients (~70%) at the time of publication. No muscular symptoms were reported for the remaining 16 cases at the time of publication, but one might anticipate that these patients will likely also develop muscular weakness later in life. Blood serum creatine kinase (CK) levels have been reported for five EBS-MD cases, with two patients showing normal and three showing increased values [18,19,20,21,22]. Electromyography (EMG) in EBS-MD patients revealed a myopathic pattern with short duration, polyphasic, and low-amplitude motor unit potentials [19,23,24,25]. Furthermore, fibrillation potentials, positive sharp waves, and pseudomyotonic/myotonic discharges have been reported, whereas nerve conduction and neuromuscular transmission appeared to be normal [19].

### 3.2. Other Skeletal Muscle-Associated Plectinopathy Disease Manifestations 

In addition to EBS-MD, skeletal muscle-related disease manifestations have been described for patients suffering from EBS-MD-MyS, EBS-PA, or LGMDR17. To date, myasthenic features in combination with EBS-MD have been reported for seven plectinopathy patients, resulting in the narration of “EBS-MD-MyS” [56,58,59,60,61]. Early onset bilateral ptosis; progressive ocular, facial, limb, and truncal weakness; and fatigability were present in all cases. In addition to muscular symptoms, which mostly appeared within the first decade of life, EBS-MD-MyS patients suffered from hypotension, alopecia totalis, hoarseness, and feeding difficulties. Significantly elevated (2–10 times) serum CK levels were reported for all patients [56,58,60,61], except for one patient with normal CK values [59]. While EMG did not show any abnormalities in a 2-year-old patient [56], it revealed a myopathic pattern in conjunction with fibrillation potentials, positive sharp waves, and complex repetitive discharges in other EBS-MD-MyS patients. Neuromuscular transmission studies using radial nerve stimulation (RNS) revealed a pathological decremental response in all patients. Tests for the presence of anti-acetylcholine receptor (AChR)- and anti-muscle-specific-kinase (MuSK)-antibodies were negative. Treatment with the cholinesterase inhibitor pyridostigmine and/or the potassium channel blocker 3,4-diaminopyridine (DAP) led to a significant improvement in some but not all EBS-MD-MyS patients. In addition, two siblings carrying a homozygous *PLEC* mutation as well as a mutation in the *CHRNE* gene, which encodes a subunit of the AChR, were described with EBD-MD-MyS [57].

Initially, for EBS-PA patients, who generally showed a more severe phenotype that included generalized skin blistering, aplasia cutis congenita, and pyloric atresia usually died within few months after birth, no signs of MD were reported [4]. However, it was reasonable to assume that these patients, if they survived longer, would have also developed MD at later points in life. In 2010, an EBS-PA patient was reported, who, based on clinical features and laboratory data, was postnatally diagnosed with MD [66]. Elevated levels of muscle enzymes, including CK and aldolase, persisted over the course of his life of three months [66]. Another case with EBS-PA experienced significant urological abnormalities and showed slightly increased CK levels at the age of 6 but demonstrated no clinical signs of MD at this age [40]. More recently, a patient with non-lethal EBS-PA and progressive MD was published [65]. This patient showed growth and motor developmental delays and started to walk and stand at the age of 20 months. CK values for this patient with EBS-MD-PA were reported for different times in life [65], with a CK peak of 1468 U/L (normal range <350 U/L) five days after birth and decreased levels over time (184 U/L and 206 U/L at 14 and 17 months of age, respectively). Since, at least in this study, the CK levels reached their peak at a very early age and before the onset of muscular symptoms, it would be advisable to also perform the corresponding measurements in plectinopathy patients at early time points.

Up until now, 13 cases with LGMDR17 due to mutations in exon 1f have been published [14,15,16]. In general, most LGMDR17 patients suffered from early onset limb-girdle syndrome followed by several years of plateau. They presented with delayed motor milestones, difficulties in walking and climbing stairs, easy fatigability, and muscle cramps. A routine EMG examination, performed on the index patient in the initial study, was clearly myopathic [14]. CK levels were markedly elevated (10- to 20-fold). Interestingly, while the first studies did not report any myasthenic features, the LGMDR17 patients presented by Mroczek et al., originating from four unrelated families, presented myasthenic symptoms such as mild ptosis from early on [15]. All four patients had high jitter in single-fiber EMG. Two patients, who had milder weakness, had a decremental response of over 10% in RNS. Moreover, two patients were reported to be negative for anti-AChR and anti-MuSK antibodies [15]. In addition to LGMDR17 caused by mutations in exon 1f, one patient suffering from severe MD with no obvious skin disorder was diagnosed with LGMDR17 due to compound heterozygous *PLEC* mutations in exons 31 and 32 (c.5995C > T, p.R1999W; c.9940T > A, p.F3314I), comparable to “classical” EBS-MD-causing *PLEC* mutations and not restricted to skeletal muscle-specific isoforms [68]. This patient developed the first signs of muscle weakness at 2 years of age (e.g., delayed independent walking, occasional falls, and difficulties climbing stairs), and muscle hypertrophy was reported at 7 years of age, including >10 times-increased serum CK levels [68]. Two more patients were diagnosed with LGMDR17 and myasthenic symptoms without any skin involvement caused by compound heterozygous *PLEC* mutations in exons 25 and 32 (c.3064C > T, p.Q1022Ter; c.11503G > A, p.G3835S), showing progressive limb and ocular muscle weakness with ptosis and dysphagia [67]. In these patients, serum CK levels were reported to be elevated, evaluation for anti-AChR antibody was negative, and EMG examinations showed a myopathic pattern, but nerve conduction studies revealed normal results [67].

### 3.3. Emerging Cardiac Pathologies in Plectinopathy Patients 

The clinical phenotypes of human plectinopathies with the predominant involvement of skin and skeletal muscle highlight the notion that plectin centrally contributes to the fundamental biomechanical properties of stress-bearing tissues. Accordingly, plectin-related disease manifestations affecting the heart, a striated muscle organ with a high mechanical workload, are indeed plausible. The concept of an importance of plectin in cardiac tissue was further substantiated by several EBS-MD case studies, which reported on cardiac disease manifestations including left ventricular hypertrophy of the heart [23], atrial fibrillation in conjunction with reduced ejection fraction and hypokinetic left ventricular cardiac walls [69], biventricular dilated cardiomyopathy [28], and left ventricular non-compaction cardiomyopathy [20]. In another EBS-MD patient, a postoperative paroxysmal atrial fibrillation after a surgery was noted [19]. In 2016, an EBS-MD patient was reported, who, in addition to the typical skin and skeletal muscle involvement, developed a dilated cardiomyopathy and life-threatening episodes of cardiac arrhythmias necessitating the implantation of a single-chamber cardioverter defibrillator [24]. This work was the first report indicating that life-threatening cardiac disease manifestations may occur before the onset of skeletal muscle symptoms, underscoring the importance of routine cardiological evaluation including electrophysiological and cardiac imaging studies that should be part of the diagnostic work-up of all EBS-MD and EBS-MD-MyS patients. This is also in line with another study, in which a family with several cases of fatal cardiomyopathy was reported, and *PLEC* mutations were finally identified for the index patient [25]. Recently, out of a family with three siblings suffering from LGMDR17 due to a mutation in exon 1f, one patient died from sudden cardiac death after spontaneous pneumothorax [16]. Finally, the role of a *PLEC* missense variant as a risk factor for atrial fibrillation has been controversially discussed [70,71]. Despite its clear clinical relevance, data on plectin in normal and diseased human hearts is very scarce. Up until now, only a single publication exists in which the pathological consequences of *PLEC* mutations on human cardiac tissue have been described [28]. In the reported EBS-MD patient with progressive biventricular cardiomyopathy due to compound heterozygous *PLEC* mutations, an aberrant plectin staining with the loss of the normal plectin-desmin colocalization at intercalated disks and Z-disks as well as a sarcoplasmic protein aggregation pathology were found.

## 4. Skeletal Muscle Biopsy Findings 

**Figure 2:**
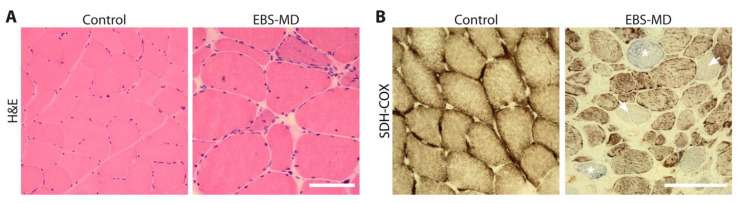
Skeletal muscle pathology in epidermolysis bullosa simplex with muscular dystrophy (EBS-MD). (**A**) Skeletal muscle sections from a healthy control and a patient suffering from EBS-MD were stained with hematoxylin/eosin (H&E). Note the rounding of the muscle fibers, the marked variation in the fiber size, the increased amounts of connective tissue and fat cells, and the clustering as well as the centralization of the myonuclei in the EBS-MD patient muscle biopsy sample. (**B**) Succinate dehydrogenase and cytochrome c oxidase (SDH-COX) double-staining of skeletal muscle sections from a healthy control and a patient suffering from EBS-MD displays altered mitochondrial morphology, including the presence of rubbed-out areas (arrows) and COX-negative fibers (*) in the EBS-MD patient but not in the control sample. Scale bars: 100 µm. (Images, courtesy of R. Schröder).

To date, detailed descriptions of skeletal muscle biopsies have been reported for 11 EBS-MD cases (see Table 2). When stained with hematoxylin/eosin (H&E), myopathological analyses of skeletal muscle specimens from EBS-MD patients revealed mild to severe myodegenerative features including increased amounts of endomysial connective and fatty tissue, rounding of the muscle fibers, and variable fiber size diameters (Figure 2A). Myopathic changes such as fiber splitting, de- and regenerating fibers, and fibers containing rimmed and non-rimmed vacuoles could be observed as well. Further, abnormal myonuclear positioning was noted, with myonuclei frequently being clustered in the subsarcolemmal region and with up to ~50% of fibers containing internally located myonuclei.

When assessed for mitochondria using succinate dehydrogenase (SDH) and cytochrome c oxidase (COX) stains, muscle specimens from EBS-MD patients demonstrated an abnormal pattern with less ordered, often coarse, and thread-like appearing mitochondrial networks. In addition, fibers with rubbed-out lesions displaying attenuated or even absent enzyme reactions as well as COX-negative fibers have been reported (Figure 2B). Immunofluorescence analyses of skeletal muscle specimens have displayed either reduced or completely absent staining for plectin, accompanied by the loss of a regularly structured desmin IF network and the occurrence of desmin-positive protein aggregates. While plectin immunoblot analysis of normal human skeletal muscle usually displays two bands, a very strong band corresponding to the ~500 kDa full-length protein and a faint signal at 390 kDa, corresponding to the rodless plectin variant, lacking the central, exon 31-encoded rod domain [72], immunoblotting analyses of EBS-MD patient-derived muscle samples reveal either the complete absence of plectin, a marked reduction in signal intensities of both bands, or the sole detection of rodless plectin [23,24,48]. Ultrastructural analyses of skeletal muscle tissues from EBS-MD patients have revealed a wide variety of changes, including the severe loss of myofibril and sarcomere organization; the loss of Z-line-plasma membrane anchorage; degenerating myofibrils, Z- and I-band alterations, autophagic vacuoles, and cytoplasmic bodies. Additionally, misplaced and degenerating mitochondria as well as the subsarcolemmal clustering of mitochondria with the occasional abnormal dumbbell shape and paracrystalline inclusions were observed. Immunogold electron microscopy revealed desmin-positive filaments and subsarcolemmal protein aggregates [23].

Skeletal muscle biopsies from EBS-MD-MyS patients displayed dystrophic features including increased variability in fiber size; the rounding of muscle fibers; internalized myonuclei, necrotic, and regenerating fibers; and an increase in fat and connective tissue. Multiple COX-negative fibers and fibers with rubbed-out lesions were reported as well [58]. Staining for plectin and desmin exhibited markedly reduced signal intensities [58]. This was in line with the virtual complete absence of plectin protein as denoted by immunoblotting analyses [58]. Ultrastructural findings demonstrated neuromuscular endplate abnormalities including the fragmentation of endplates, the destruction of the junctional folds, and postsynaptic simplification [57]. Myofibrils appeared disarranged and disrupted. Moreover, large accumulations of heterochromatic and lobulated nuclei, including rare apoptotic nuclei, were observed. Mitochondria displayed pathological changes, and subsarcolemmal and intrafiber clusters of mitochondria became apparent [60,61].

Histopathological analyses of skeletal muscle from patients with P1f-associated LGMDR17 demonstrated severe dystrophic features with variation in fiber size, internal nuclei, scattered basophilic and necrotic fibers, mild endomysial fibrosis, a swollen endomysium, and predominance of type 2 fibers [14,15]. Electron microscopy revealed dystrophic and destructive changes, focal disorganization of the myofibrils, enlarged space between the membrane and sarcomeres, Z-line disorganization, sarcolemmal duplications, and dilation of the tubular system. In addition, degenerating mitochondria and subsarcolemmal and intra-fiber clusters of mitochondria were reported [15,16]. Immunofluorescence analyses of plectin demonstrated its loss in the muscle fiber sarcolemma, while sarcoplasmic plectin-staining was still preserved, indicating the presence of isoforms other than P1f [14,16]. Accordingly, RT-PCR analysis revealed a complete absence of P1f-specific mRNA in a patient muscle sample, while the total *PLEC* mRNA was detectable [14].

## 5. Muscle-Related Molecular Pathology 

### 5.1. Downstream Effects of Human PLEC Mutations on Skeletal Muscle Organization

**Figure 3:**
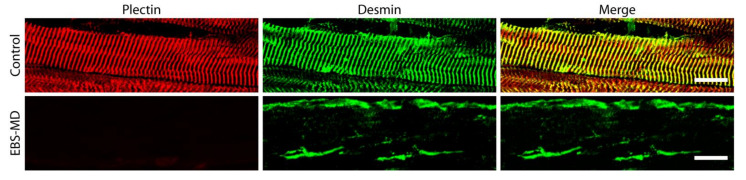
Immunofluorescence microscopy of longitudinal cryosections from healthy control and EBS-MD skeletal muscle specimens using antibodies to plectin (red labelling) and desmin (in green). Note the striated pattern and co-localization of plectin and desmin in the control muscle as well as the absence of plectin staining and the massive aggregation of desmin intermediate filaments (IFs) in the EBS-MD muscle. Scale bars: 20 µm. (Image of EBS-MD patient, courtesy of R. Schröder).

**Figure 4:**
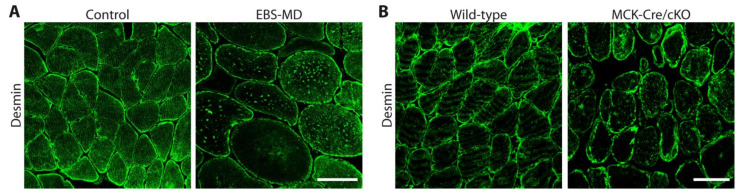
Immunofluorescence staining for desmin (in green) of cross sections from frozen skeletal muscle samples from a healthy control in comparison to an EBS-MD patient (**A**), and from a wild-type mouse in comparison to the conditional muscle-specific MCK-Cre/cKO mouse (**B**), which reveals the disruption of the desmin IF network and the formation of desmin-positive subsarcolemmal and cytoplasmic protein aggregates in plectin-deficient muscles. Scale bars: 100 µM (**A**), and 50 µM (**B**). (Images in (**A**), courtesy of R. Schröder).

One might anticipate plectin’s influence on IF network organization and function to be the most prominent in skeletal muscle fibers, which, comparable to the case of desmin deficiency, lose their structural integrity in its absence [3]. However, even though the skin blistering phenotype in plectinopathy patients has been intensively investigated and was attributed to a disruption of the normal keratin IF anchorage to hemidesmosomes on the molecular level (for review see [73]), the downstream effects of human *PLEC* mutations in EBS-MD skeletal muscle are less clearly defined, and to date, they have only been addressed in very few detailed patient descriptions [23,24,28,48,49]. In general, a severe disruption of the extrasarcomeric IF cytoskeleton was observed, resulting in a drastic reduction or a complete loss of the characteristic cross-striated desmin staining pattern in longitudinal sections of EBS-MD muscle (Figure 3), while cross sections revealed the accumulation of cytoplasmic and subsarcolemmal desmin aggregates (Figure 4A) [23,24,48]. In a recent study, skeletal muscle biopsies from three different EBS-MD cases were comparatively evaluated, resulting in the visualization of protein aggregates containing desmin, syncoilin, and synemin, indicating that plectin is the key cytolinker protein regulating the structural and functional organization of the skeletal muscle IF cytoskeleton [24]. The loss of a functional extrasarcomeric IF network ultimately leads to degenerative myofibrillar changes, Z-disk alterations and displacement, and disrupted Z-line-plasma membrane anchorage, as visualized for several EBS-MD patients using electron microscopy [22,24,26,48,49]. 

Mitochondrial abnormalities reported for EBS-MD patients comprise altered organelle distribution, massive subsarcolemmal aggregation of mitochondria, and reduced total amounts of organelles [19,23,24,26]. Comparative immunoblotting analyses using antibodies directed against complex II and V of respiratory chain enzymes showed a substantial decrease in the respective protein levels in three different EBS-MD patients [24]. Moreover, the biochemical analyses of the skeletal muscle homogenates of an EBS-MD patient carrying a 16-bp insertion mutation close to the IF-binding site revealed substantial respiratory chain dysfunctions, such as decreased complex I or COX activities [23]. In EBS-MD-MyS patients, the subsarcolemmal clustering of mitochondria was reported, leaving other fiber areas depleted of mitochondria [61]. Similarly, for a EBS-MD-MyS patient carrying homozygous *PLEC* and *CHRNE* mutations, an accumulation of thread-like mitochondria at the neuromuscular junctions (NMJs) was reported [57]. In conclusion, by exhibiting sarcoplasmic and subsarcolemmal desmin-positive protein aggregates, degenerative changes of the myofibrillar apparatus, and mitochondrial abnormalities, most skeletal muscle-related plectinopathies can be annotated among the expanding group of myofibrillar myopathies (MFMs), a numerically significant subgroup of hereditary and sporadic protein aggregate myopathies [4,74,75]. MFMs are caused by mutations in genes coding for various sarcomeric and extrasarcomeric proteins, such as desmin (*DES*), *PLEC*, filamin C (*FLNC*), myotilin (*MYOT*), αB-crystallin (*CRYAB*), ZASP (*ZASP*), BAG3 (*BAG3*), FHL1 (*FHL1*), titin (*TTN*), α-actin (*ACTA1*), HSPB8 (*HSPB8*), and DNAJB6 (*DNAJB6*) [74]. All MFMs are progressive and devastating diseases of the human skeletal muscle that often lead to premature death. To date, no causative or ameliorating therapy is available for this significant cohort of hereditary myopathies. 

### 5.2. Muscle-Associated Molecular Pathomechanisms in Plectinopathy Animal and Cell Models

Molecular studies on human muscle-related plectinopathies are generally aggravated by the very limited amount of patient muscle samples, the fact that the alterations noticed in these specimens usually reflect late stages of the disease, and the highly heterogeneous origin regarding age, sex, biopsied muscle, or disease severity. Therefore, to investigate the highly complex disease pattern of human plectinopathies, corresponding cell and animal models closely mimicking the human disease pathology are necessary. 

Plectin knock-out (KO) mice, lacking all full-length and rodless plectin variants, displayed various necrotic changes, including the loss of myofilaments and the focal disruption of sarcomeres affecting Z-lines and adjacent myofibrils, when assessed by electron microscopy [76]. Interestingly, in the affected areas, multiple structural abnormalities such as focal disintegration, loss of cross-striations, and Z-band streaming appeared, reminiscent of minicore myopathies [76,77]. Additionally, swollen mitochondria and aggregated organelles in the autolysis stage were detected in the damaged fibers [77]. In plectin-deficient cardiac muscle, the partial disintegration of intercalated disks and adjacent myofibers was noted [76]. However, as an animal model for investigating the type of late-onset muscular dystrophy that is characteristic of most plectinopathies, such mice are of limited use, as they die within 2–3 days after birth because of internal blistering of the oral cavity that disallows food intake. 

As the majority of EBS-MD-causing mutations occur within exon 31, which encodes the central rod domain, leaving the generation of a low-level rodless plectin splice variant unaffected, corresponding rodless plectin mice were generated [78]. Interestingly, the rodless plectin mice were viable and fertile and did not show any signs of skin blistering or muscular dystrophy, indicating that rodless plectin can functionally compensate for full-length plectin and that in EBS-MD patients, the overall rather low expression or complete loss of plectin rather than the absence of the rod domain is crucial for disease development [78].

**Figure 5:**
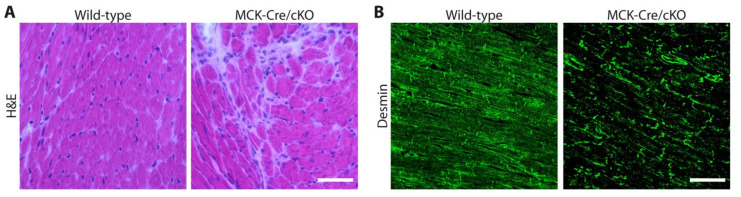
Heart sections from wild-type and MCK-Cre/cKO mice were stained with H&E (**A**) or subjected to immunofluorescence microscopy using anti-desmin antibodies (in green) (**B**). Note the increased connective tissue formation in H&E-stained MCK-Cre/cKO sections, which is indicative of cardiomyocyte degeneration. Additionally, note the focal disruption of the contractile apparatus and the desmin aggregation in the MCK-Cre/cKO cardiomyocytes. Scale bars: 50 µM (**A**) and (**B**).

To specifically investigate plectin’s role in skeletal muscle in more detail, plectin floxed mice were bred to muscle creatine kinase (MCK)-Cre mice expressing the Cre transgene under the control of the MCK promoter, thereby generating conditional plectin KO mice (MCK-Cre/cKO) with plectin deficiency restricted to skeletal muscle and the heart [9]. Starting from 6 months of life, the MCK-Cre/cKO mice exhibited decreased motor endurance performance and survival rates. H&E-stained skeletal muscle sections from the MCK-Cre/cKO mice revealed numerous necrotic, hypertrophic and split fibers, centralized myonuclei, and increased amounts of connective tissue. While a quantification of the cross-sectional areas of fibers revealed high fractions of hypertrophic and small-diameter fibers, the total number of fibers was slightly increased in the MCK-Cre/cKO soleus muscles [9]. Showing progressive degenerative alterations in striated muscle, including the misalignment of the Z-disks, the detachment of the contractile apparatus from the sarcolemma, desmin IF network collapse, and pathological protein aggregation, MCK-Cre/cKO mice closely mimicked the pathology of EBS-MD patients (Figure 4B) [9]. These mice also provided experimental evidence to show that the lack of plectin also leads to severe cardiac tissue alterations. The hearts of 16-month-old cKO mice displayed marked cardiac fibrosis (Figure 5A) [9] and a disorganization of the myofibrillar apparatus as well as the above described desmin aggregate pathology (Figure 5B). In addition, abnormalities in mitochondrial morphology and function were reported in MCK-Cre/cKO muscle. Mitochondria were significantly reduced, were no longer associated with the Z-disks, and were massively aggregated in the sarcoplasmic and subsarcolemmal regions. Decreased mitochondrial citrate synthase activity, respiratory function, and adenosine diphosphate kinetics were also observed in plectin-deficient muscles [79]. Finally, profound changes in the costameric lattice were observed in MCK-Cre/cKO muscles; not only did the signals of syncoilin and synemin, originally attributed to linking desmin IFs to the sarcolemma and Z-disks, co-localize with desmin-positive aggregates, but they also co-localized with cytokeratin IFs and components of the dystrophin–glycoprotein complex (DGC), confirming plectin’s crucial role as a skeletal muscle IF network organizer [9].

To analyze skeletal muscle-specific pathological mechanisms on a single cell level, immortalized plectin-deficient (*Plec^−/−^*) myoblast cell lines were established from plectin-null mice that had been crossed into a *p53^−/−^* background, an experimental strategy to overcome the Hayflick limit of primary myoblasts and to obtain large quantities of cells for biochemical or immunoblotting analyses [80]. Similar to plectin-deficient primary myoblasts, immortalized *Plec^−/−^* myoblasts of this type differentiated into mature, spontaneously contracting, multinucleated myotubes that closely mirrored the pathology of plectinopathy patients, including the formation of desmin aggregates and a concurrent disarrangement of the myofibrillar apparatus [80]. Since desmin appears to assemble into filaments normally in *Plec^−/−^* myoblasts, plectin does not seem to be required for IF network formation per se but has an essential role in the proper attachment of IFs to the myofibrillar apparatus and to other structures, such as the subsarcolemmal and nuclear cytoskeleton. Accordingly, as demonstrated by subcellular fractionation, mitochondria are less firmly bound to the cytoskeleton, and desmin IFs become more soluble in the absence of cross-linking and anchorage through plectin in *Plec^−/−^* cell lysates compared to the *Plec^+/+^* controls [79]. Moreover, the mitochondrial networks in *Plec^−/−^* myoblasts and differentiated myotubes appeared enlarged [79]. Analyses of protein dynamics inside living myotubes by fluorescence recovery after photobleaching (FRAP) revealed the increased molecular mobility of (not aggregate-localized) desmin and the Z-disk marker protein α-actinin in *Plec^−/−^* cells [80]. In addition, biomechanical properties, such as cellular stiffness, as assessed using a magnetic tweezer device, strain energy, or adhesion strength as well as response to mechanical stress were significantly reduced in plectin-deficient myoblasts [81]. Taken together, these studies highlight plectin’s role as a crucial IF network stabilizing element, as IFs that are not anchored via plectin at subcellular docking sites become destabilized, more mobile, and presumably more accessible to posttranslational modifications, which, in turn, make them more prone to aggregation.

In patients suffering from EBS-MD-MyS, a grossly disorganized NMJ morphology became apparent. In plectin-deficient differentiated myotubes, reduced amounts of AChR clusters were formed upon exposure to agrin, and the ones that had formed appeared highly mobile and were unable to coalesce into stable clusters [82]. To investigate the effect of plectin on NMJ architecture in an animal model, conditional plectin KO mice with gene disruption in muscle precursor (satellite) cells (Pax7-Cre/cKO) were generated [82], resulting in plectin deficiency in the satellite cells and consequently in mature muscle cells and also at the NMJs, which is in contrast to the MCK-Cre/cKO mice, where satellite cells and NMJs were exempt from ablation. Pax7-Cre/cKO mice suffered from body weakness, which manifested as small size, profound kyphosis, and a decreased survival rate of only 50% at the age of 19 weeks [82]. Moreover, the uncoupling of the AChRs from the postsynaptic desmin IF network led to the loss of membrane infoldings and to the disorganization of the NMJ microenvironment, including its invasion by microtubules, ultimately leading to impaired body balance and severe muscle weakness in Pax7-Cre/cKO mice, closely mimicking the clinical disease manifestations of EBS-MD-MyS patients. In conclusion, it has been shown that plectin mediates the linkage of AChR complexes to the desmin IF network and that this connection is crucial for endplate integrity and proper body locomotion [82].

## 6. Skeletal Muscle-Expressed Pectin Isoforms as the Key to Desmin IF Network Architecture

**Figure 6:**
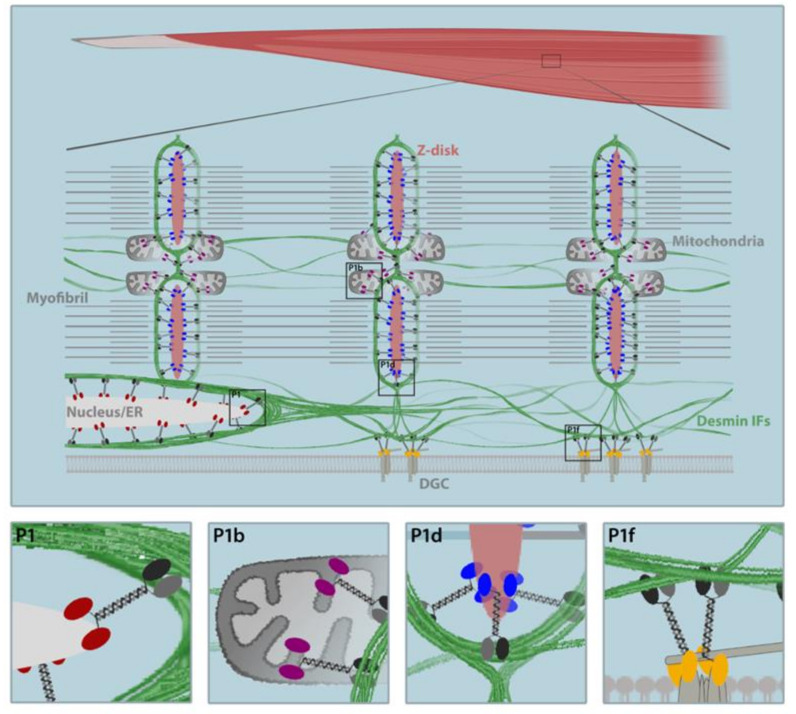
Scheme depicting plectin’s versatile function in skeletal muscle. Distinct localization and desmin IF-anchoring functions of plectin isoforms, determined by the differentially spliced N-terminal exons, are highlighted: plectin isoform 1 (P1, red) targets desmin IFs to the outer nuclear/endoplasmic reticulum (ER) membrane system, P1b (purple) targets them to the mitochondria, P1d (blue) to the Z-disks, and P1f (yellow) targets them to the costameric lattice/dystrophin–glycoprotein complex (DGC). As such, by interlinking neighbouring myofibrils and cytoplasmic organelles with the sarcolemma, plectin provides stability to the extrasarcomeric cytoskeleton.

Analysis of the plectin gene revealed a complex organization of close to 50 exons spanning over >62 kb of DNA and an unusual variety of isoforms containing alternative first coding exons, which are spliced into a common exon 2. Various plectin isoforms, only differing in their very N-terminal sequences, form the basis for plectin’s broad versatility, as they determine their subcellular organization and secure their tissue-specific expression [7]. Thus, different cell types and tissues vary from each other in the composition and proportion of the plectin isoforms that are expressed. All eight of the originally identified transcript variants containing differentially spliced coding first exons (P1–P1g) were found to be expressed in skeletal muscle; although for some plectin isoforms, very weak signals were detected by RNase protection assay [6]. In this study, a tissue-specific P1d dominance was detected, which was prevailing in skeletal muscle and in the heart. Beyond P1d, the skeletal muscle set primarily comprised P1f, P1b, and P1, with relative mRNA ratios of >10:4:3:1 for P1d, P1f, P1b, and P1 [6], and every isoform is specifically targeted to a distinct subcellular location [7,8,9]. There, the four skeletal muscle-specific plectin isoforms fulfill different functions and orchestrate the skeletal muscle-specific desmin IF network cytoarchitecture (see Figure 6). Isoform-specific KO mice, lacking just one specific plectin variant while expressing all of the others, and the cell cultures derived from them, enabled a detailed investigation of the functional and molecular consequences of plectin isoform ablation.

### 6.1. P1d Docks Desmin IFs to the Sarcomere

As determined by RNase protection mapping, P1d is the only plectin isoform that is exclusively expressed in skeletal muscle and in the heart [6]. Even though the isoform-specific N-terminal sequence of P1d is only five amino acids long, it targets P1d-GFP-fusion proteins exclusively to the Z-disks of differentiated myotubes [8]. When the skeletal muscle fibers from P1d-KO mice were immunolabeled using pan-plectin antibodies (reactive with all plectin isoforms), only sarcolemmal staining and dotty remnants in the interior of the fiber became evident, indicating that the peripheral, perinuclear, and costameric regions, harboring P1 and P1f, were still preserved [9]. Consequently, P1d-deficiency resulted in the aggregation of desmin IFs in the interior but not in the peripheral areas of skeletal muscle fibers. In addition, mitochondrial alterations were observed in P1d-deficient muscles, including the altered distribution and subsarcolemmal aggregation of mitochondria, compromised respiratory activity, and mitochondrial lysis [9,79]. Including the misalignment of myofibrils and the disorientation of Z-disks, cryosections derived from P1d-KO muscles revealed a pathology closely resembling that of MCK-Cre/cKO muscles at the histochemical level [9,79]. However, compared to the latter, in P1d-deficient muscle fibers, the desmin aggregates were smaller and the mitochondria were often found in the close vicinity of the desmin network remnants and the Z-disks, indicating that mitochondria-associated P1b was still able to interlink the desmin IFs and the organelles [79]. Likewise, the IF network underneath the sarcolemma appeared unaffected in P1d-KO teased fibers, suggesting that P1f still maintains the organization of the costameres [9]. In plectin-deficient differentiated myotubes, which displayed impaired Z-disk alignment and desmin IF network collapse, the forced expression of full-length P1d could fully restore sarcomere formation and recruited desmin IFs to the Z-disks [80]. In contrast, the forced expression of any of the other skeletal muscle-specific plectin isoforms (P1, P1b, or P1f) showed no such effect, indicating that the striking sarcomere pathology in EBS-MD muscle is primarily due to a loss of function of isoform P1d, which, in partnership with desmin IFs, cross-links individual myofibrils to each other at the Z-disk level.

### 6.2. P1f Tethers Desmin IFs to the Sarcolemma

The forced expression of GFP-tagged P1f in differentiated myotubes showed that the fusion protein was exclusively targeted to the sarcolemma [8]. In teased muscle fibers, P1f was strongly expressed at the Z-disks, tightly encircled the nuclei, and was associated with the DGC at the sarcolemma [8]. As plectin directly interacts with dystrophin and the transmembrane laminin receptor β-dystroglycan, two of the major DGC constituents, via multiple binding sites in its N-terminal region, leaving its C-terminal IF-binding site freely accessible [8], it is capable of docking the desmin IF network to the membrane. Thus, P1f connects costameres with peripheral myofibrils, mitochondria, and myonuclei. In addition, P1f is required for NMJ integrity in fully developed myofibers, where AChR-rapsyn complexes, positioned at the crests of postsynaptic folds, are physically linked via rapsyn-bound P1f to the desmin IF network [82]. Moreover, a striking similarity of the temporal expression of P1f and dystrophin during the differentiation of myoblasts to multinucleated myotubes suggests a role of P1f during the formation and maturation of costameres [8], which is comparable to the transformation of focal adhesions (FAs) to fibrillar adhesions observed in fibroblasts [83]. This is supported by the observation that during the differentiation of human myoblasts, plectin is primarily a component of longitudinal adhesion structures, which are precursors of costameres and only mature after being subjected to contractile forces [84,85]. As P1f was additionally localized to the Z-disks in certain muscle fiber types, one might anticipate a certain degree of interchangeability of certain plectin isoforms once the IF-docking sites have been formed [8]. In conclusion, myofiber integrity is preserved by a desmin IF-mediated structural axis generated by P1d and P1f, interlinking the sarcomeres at the Z-disks and tethering them to the plasma membrane. Accordingly, skeletal muscle specimens from LGMDR17 patients revealed a loss of sarcolemmal (P1f) but not sarcoplasmic plectin staining (P1d), which was accompanied by an corresponding accumulation of desmin aggregates [16], while the elimination of P1d in mice leads to the formation of desmin aggregates in the interior of the fiber but not in its peripheral sarcolemmal region [9].

### 6.3. P1b Links Desmin IFs to the Mitochondrial Network

Isoform P1b co-distributes with mitochondria while also being associated with IFs, thus likely forming a bridge between the filament network and the organelles [7,86]. The exon 1b-encoded sequence serves as a mitochondrial targeting and anchoring signal, inserting the protein via its N-terminal part into the outer mitochondrial membrane, whereas the bulk of the protein remains in the cytosol, where it recruits IFs via its C-terminal binding site [86]. The loss of P1b leads to marked mitochondrial shape changes, manifesting as the elongation of the mitochondrial networks in P1b-deficient fibroblasts and myoblasts [86]. In muscle fibers that are deficient for P1d (but not P1b), the association of residual plectin with mitochondria and the colocalization of collapsed desmin IFs with organelle aggregates were reported, indicating that mitochondria-associated P1b was still able to fulfill its function as a linker between the (residual) IF network and mitochondria [9,79]. This was neither the case in P1b-KO nor in MCK-Cre/cKO muscle. On the other hand, in P1b-KO muscle, the structural integrity of the myofibers seemed to be maintained, as other isoforms (P1d) were still interlinking the contractile apparatus via the desmin IF cytoskeleton (and P1f) to the sarcolemma [79]. Thus, in P1b-KO muscles, the mitochondria were properly arranged along Z-disks, while their lateral alignment appeared less tight and regular, suggesting a loss of their P1b-mediated anchorage to the IF network [79]. Consequently, permeabilized muscle fibers isolated from the heart, soleus, and gastrocnemius muscles of the P1b-KO mice displayed pronounced mitochondrial dysfunctions, including decreased mitochondrial citrate synthase activity, 2- to 3-fold reduced respiratory capacities, and significantly decreased adenosine diphosphate kinetics compared to wild-type tissues [79]. Whether the elongation of mitochondrial networks and their functional impairments directly correlate with the observed increased protein levels of mitochondrial fusion-related protein mitofusin-2 (Mfn-2) in P1b-KO muscle lysates [79] remains to be elucidated. In conclusion, P1b affects the organelle shape by tethering mitochondria to IFs and presumably forms a mitochondrial scaffold for proteins that establish and/or maintain mitochondrial morphology at the surface of the organelle. Another possibility would be that the interlinkage of mitochondria and IFs by P1b plays a mechanical role in shaping the organelle.

### 6.4. P1 Connects Desmin IFs to the Nucleus/ER Membrane System

The isoform-specific N-terminal sequence of P1 is the longest of all of the plectin isoforms (180 amino acid residues) and contains a KKDRR sequence, which constitutes a classical monopartite nuclear localization signal (NLS) [6,7]. Therefore, truncated N-terminal P1 fragments up to a certain length are localized in the nucleus [7]. In skeletal muscle fibers, P1 is primarily targeted to the outer nuclear/endoplasmic reticulum (ER) membrane system, likely via interaction with endophilin B1/B2, where it orchestrates the perinuclear desmin IF network organization [9,87]. Accordingly, in teased muscle fibers from P1-KO mice, the perinuclear desmin networks collapsed and aggregated, ultimately affecting nuclear morphology and function. Myonuclei in muscle fibers from P1-KO (as well as MCK-Cre/cKO) mice were bigger and more rounded, irregularly spaced, and frequently clustered [87]. P1-deficiency in skeletal muscle ultimately affected chromatin modifications and the expression of various genes, including signaling pathways mediating mechanotransduction, metabolism, and stress response [87]. In addition to the nuclear/ER membrane system, P1 showed partial association with the sarcolemma in certain fiber types [8]. Thus, comparable to P1f, P1 may show partial interchangeability with other isoforms at the mature or late stages of muscle development.

## 7. From Cell and Animal Models to Potential Therapeutic Approaches

Understanding the early molecular and progressive mechanisms that determine the pathology of plectinopathies as well as other types of MFMs is a prerequisite for the development of potential therapeutic approaches. Cell and animal models closely recapitulating the human pathology are inevitable for both deciphering molecular pathomechanisms and assessing novel treatment concepts. As described herein, studies on EBS-MD patient samples, MCK-Cre/cKO mice, or plectin-deficient myotubes demonstrated that different *PLEC* mutations, irrespective of their individual consequences on plectin protein expression, cause a defective structural and functional organization of the extrasarcomeric desmin cytoskeleton, which triggers the formation of desmin protein aggregates in skeletal muscle tissue. As a consequence, the severe derangement of the desmin cytoskeleton leads to the faulty alignment and anchorage of myofibrils and secondary mitochondrial abnormalities comprising respiratory chain dysfunction and altered organelle distribution and amount. However, additional pathomechanisms such as altered protein degradation processes likely contribute to these effects. In fact, increased protein levels of various heat shock proteins (HSPs), molecular chaperones that assist in the establishment of proper protein conformation and that prevent the aggregation of partially denaturated proteins [88], were observed in skeletal muscle specimens from MCK-Cre/cKO mice and, to some extent, also during the differentiation of *Plec^−/−^* myotubes, indicating that chaperones might represent a first line of defense against desmin network aggregation [80]. As low-molecular-weight chaperones, similar to HSPs, have the capacity to prevent protein aggregation and to contribute to the rescue of in vivo aggregated proteins [89]; such substances appear to be a promising attempt for alleviating aggregate formation. In fact, the treatment of *Plec^−/−^* myotubes with the chemical chaperone 4-phenylbutyrate (4-PBA) has already been approved for clinical use in patients with urea cycle disorders, resulting in the reduced formation of desmin-positive aggregates and increased sarcomere stability [80]. Furthermore, 4-PBA treatment of MCK-Cre/cKO mice achieved a marked reduction of desmin aggregate load, as observed in skeletal muscle specimens, and led to a functional muscle improvement, as assessed by grip strength measurements [80]. Evidently, 4-PBA cannot restore plectin-mediated desmin IF anchorage but probably stabilizes the filament networks within the myofiber. However, some adverse effects on EBS have been observed upon 4-PBA treatment of *KRT5* and *KRT14* mutant keratinocytes [90], indicating a complex interplay of benefits and disadvantages that requires additional research that challenges the use of 4-PBA in plectinopathy patients. Nonetheless, these results highlight the importance of plectinopathy cell and animal models for the screening and testing of pharmacological compounds, laying the basis for future therapeutic approaches.

## 8. Conclusions and Future Directions

Taken together, the muscle-specific clinical phenotypes of individual plectinopathy patients vary considerably with regard to the onset and progression of muscular symptoms and the severity and amounts of additional disease manifestations. The cause of the clinical variability is not yet fully understood and can only partially be explained by the location of the mutations in different domains and in various isoforms. Specifically, milder defects, such as myasthenic features in some cases, might have been underestimated and patients with *PLEC* mutations and should be thoroughly examined. Moreover, regarding undiagnosed muscular dystrophies and neuropathies, additional plectin mutations (including isoform-specific mutations) can be expected to be identified in increasing numbers over the next few years.

On the molecular level, plectin has emerged as the major networking and anchoring element of the skeletal muscle extrasarcomeric desmin IF network and is therefore crucial for muscle fiber architecture and integrity. By interlinking the desmin filament network throughout the extrasarcomeric space, plectin connects neighboring myofibrils to each other at the Z-disk level and to the sarcolemma and integrates mitochondria, nuclei, and other organelles into this network. In the absence of plectin, the muscle loses its integrity and the myofiber cytoarchitecture is profoundly changed. However, the knowledge of the precise molecular mechanisms and signaling events that translate plectinopathy-causing gene mutations into the myopathic phenotype is still limited but critical for the understanding of patient needs and the development of treatment concepts. Additional molecular mechanisms such as altered signaling pathways and impaired protein degradation mechanisms, including autophagy, are supposed to contribute to the observed pathology. Plectinopathy mouse models and ex vivo cell cultures will provide essential tools for the further elucidation of the molecular mechanisms leading to skeletal muscle pathology.

## Figures and Tables

**Table 1:** Patients with genetically determined *PLEC* mutations associated with muscle-related disease manifestations.

Ref	Mutation 1		Mutation 2		Geno-	MD	Sex	MB
	DNA	Protein	DNA	Protein	Type	(Onset)		
**EBS-MD**								
[25]	906 + 19_40del *	V303_P313ins11	906 + 19_40del *	V303_P313ins11	hom.	adolescence	F	no
[26]	954_956dupGCT	L319dup	4222C > T	Q1408X	c.het.	MD not dev. at 4 years; but histological changes	M	yes
[26]	954_956dupGCT	L319dup	4222C > T	Q1408X	c.het.	N/A	M	no
[27]	956T > C	L319P	2807G > A	W936X	c.het.	MD not dev. at 18 years	M	no
[27]	956T > C	L319P	6955C > T	R2319X	c.het.	MD not dev. at 31 years	F	no
[28]	968G > A	R323Q	4840G > T	E1614X	c.het.	twenties	M	yes
[28]	968G > A	R323Q	4840G > T	E1614X	c.het.	MD not dev. at 9 years	F	no
[29]	1530_1531ins36	A510_I511ins12	2677_2685del	Q893_A895del	c.het.	42 years	F	N/A
[30]	1648C > G	R500G	1648C > G	R500G	hom.	MD not dev. at 2 years	M	no
[21]	2264_2266del	F755del	2264_2266del	F755del	hom.	twenties	F	n.s.
[24]	2264_2266del	F755del	3119_3210del	K1040RfsX	c.het.	27 years	M	yes
[31]	2264_2266del	F755del	9194dup	S3066EfsX55	c.het.	MD not dev. at 3 years	M	no
[32,33]	2677_2685del	Q893_A895del	2677_2685del	Q893_A895del	hom.	early thirties	F	no
[32,33]	2677_2685del	Q893_A895del	2677_2685del	Q893_A895del	hom.	early thirties	F	no
[19]	2677_2685del	Q893_A895del	4930C > T	Q1644X	c.het.	28 years	M	yes
[34]	2694−9_2705del	N/A	5032delG	V1678WfsX65	c.het.	MD not dev. at 5 months	F	no
[33,35,36]	3157C > T	Q1053X	5806C > T	Q1936X	c.het.	infancy	M	no
[37]	3341 + 1G > T	N/A	6955C > T	R2319X	c.het.	MD not dev. at 1.5 years	-	no
[37]	4126−4A > G	N/A	7804C > T	Q2602X	c.het.	18 years	-	no
[37]	4216C > T	Q1421X	4216C > T	Q1421X	hom.	teens	-	no
[38]	4294_4306dup	V1436GfsX40	4365delC	S1456RfsX93	c.het.	20 years	M	N/A
[36,39]	4348C > T	Q1450X	4348C > T	Q1450X	hom.	19 years	F	no
[40]	4549C > T	R1517X	4549C > T	R1517X	hom.	MD not dev. at 24 years	M	no
[36]	4643_4667dup	K1558GfsX89	7120C > T	Q2374X	c.het.	MD not dev. at 7 years	M	no
[41]	4840G > T	E1614X	4840G > T	E1614X	hom.	teens	-	no
[24,42]	5018_5036del	L1673RfsX64	5018_5036del	L1673RfsX64	hom.	MD not dev. at 5 years	F	yes
[43]	5105_5112del	R1702QfsX14	5105_5112del	R1702QfsX14	hom.	10 years	M	n.s.
[44]	5137C > T	Q1713X	7051C > T	R2351X	c.het.	MD not dev. at 4 years	M	no
[45]	5254C > T	Q1752X	7285C > T	R2429X	c.het.	adolescence	F	n.s.
[18]	5257dupG	E1753GfsX17	5257dupG	E1753GfsX17	hom.	MD not dev. at 3 years	F	no
[46]	5410G > T	E1804X	5410G > T	E1804X	hom.	17 years	M	no
[46]	5410G > T	E1804X	5410G > T	E1804X	hom.	15 years	M	n.s.
[47,48]	5728C > T	Q1910X	5728C > T	Q1910X	hom.	infancy	F	yes
[47,48]	5728C > T	Q1910X	5728C > T	Q1910X	hom.	infancy	F	n.s.
[37]	5770C > T	Q1924X	N/A	N/A	N/A	30 years	-	no
[32,33,36,49]	5815delC	L1939WfsX6	5815delC	L1939WfsX6	hom.	late twenties	F	yes
[50]	5849_5856dup	E1953WfsX8	5849_5856dup	E1953WfsX8	hom.	infancy	M	n.s.
[42,49]	5854_5855del	E1952GfsX60	5854_5855del	E1952GfsX60	hom.	MD not dev. at 3 years	F	yes
[22]	5902_5093del	K1968GfsX44	9109_9125del	V3037CfsX78	c.het.	8 years	M	n.s.
[34]	6013G > T	E2005X	13378A > T	K4460X	c.het.	MD not dev. at 6 months	M	no
[51]	6622C > T	Q2208X	8119C > T	Q2707X	c.het.	6 years	M	no
[36]	6549_6582del	L2184RfsX21	13040dupG	I4348HfsX8	c.het.	10 years	F	no
[37]	6682C > T	Q2228X	10456C > T	Q3486X	c.het.	5 years	-	no
[52]	6955C > T	R2319X	6955C > T	R2319X	hom.	25 years	F	no
[20]	7100C > T	R2351X	7100C > T	R2351X	hom.	teens	M	no
[53]	7159G < T	E2387X	7159G < T	E2387X	hom.	adolescence	F	no
[33,35,36]	7261C > T	R2421X	12578_12581dup	Y4195DfsX41	c.het.	5 years	M	no
[41]	7261C > T	R2421X	N/A	N/A	N/A	childhood	-	no
[41,49,50]	7393C > T	R2465X	7393C > T	R2465X	hom.	early childhood	M	yes
[54]	7468C > T	Q2490X	7468C > T	Q2490X	hom.	MD not dev. at 4 years	M	no
[55]	10909C > T	R3637C	10909C > T	R3637C	hom.	yes (onset N/A)	M	no
[55]	10909C > T	R3637C	10909C > T	R3637C	hom.	yes (onset N/A)	M	no
[23,24]	13459_13474dup	E4492GfsX48	13459_13474dup	E4492GfsX48	hom.	4 years	F	yes
**EBS-MD-MyS**								
[56]	IVS11 + 2T > G	N/A	10187_10190del	K3395GfsX11	c.het.	birth	M	yes
[57]	1500_1501ins36	R500_V501ins12	1500_1501ins36	R500_V501ins12	hom.	childhood	F	yes
[57]	1500_1501ins36	R500_V501ins12	1500_1501ins36	R500_V501ins12	hom.	childhood	M	no
[58]	2539−2A > G	N/A	11737delC	R3913VfsX30	c.het.	25 years	M	yes
[59]	3086G > A	R1029H	9679_9766del	D3229VfsX21	c.het.	N/A	F	no
[59]	3086G > A	R1029H	9679_9766del	D3229VfsX21	c.het.	N/A	M	no
[59]	3086G > A	R1029H	9679_9766del	D3229VfsX21	c.het.	N/A	M	no
[60,61]	6169C > T	Q2057X	12043dupG	E4015GfsX69	c.het.	9 years	F	yes
[61]	6955C > T	R2319X	12043dupG	E4015GfsX69	c.het.	3 years	M	yes
**EBS-PA**								
[62]	913C > T	Q305X	913C > T	Q305X	hom.	N/A	M	no
[63]	913C > T	Q305X	1344G > A	N/A	c.het.	N/A	M	no
[62]	1563_1567del	G522WfsX11	1563_1567del	G522WfsX11	hom.	N/A	F	no
[64]	2680_2693del	E894AfsX84	2680_2693del	E894AfsX84	hom.	N/A	F	no
[62]	2769_2788del	W923CfsX53	2769_2788del	W923CfsX53	hom.	N/A	M	no
[40]	2888dupT	F963PfsX19	N/A	Q2367X	c.het.	MD not dev. at 6 years	F	no
[37]	3342−2A > G	N/A	3902_3903del	Q1301LfsX8	c.het.	N/A	-	no
[63]	3565C > T	R1189X	3565C > T7612C > T	R1189XQ2538X	hom.& c.het	N/A	F	no
[37]	4119_4120del	N/A	12499C > T	R4167X	c.het.	MD not dev. at 12 years	-	no
[39]	7396C > T	Q2466X	7633C > T	Q2545X	c.het.	N/A	M	no
[62]	9085C > T	R3029X	9085C > T	R3029X	hom.	N/A	F	no
[65]	11912del	K3971Ter	12499C > T	R4167X	c.het.	birth	M	no
[66]	10984C > T	E3662X	11453_11462del	I3818RfsX90	c.het.	birth	M	no
**LGMDR17** (**P1f mutation**)								
[14]	1_9del **	-	1_9del **	-	hom.	3 years	M	yes
[14]	1_9del **	-	1_9del **	-	hom.	early childhood	M	no
[14]	1_9del **	-	1_9del **	-	hom.	early childhood	F	no
[14]	1_9del **	-	1_9del **	-	hom.	early childhood	F	no
[14]	1_9del **	-	1_9del **	-	hom.	2 years	M	yes
[14]	1_9del **	-	1_9del **	-	hom.	early childhood	M	n.s.
[15]	1_9del **	-	1_9del **	-	hom.	6 years	F	n.s.
[15]	1_9del **	-	1_9del **	-	hom.	26 years	F	n.s.
[15]	1_9del **	-	1_9del **	-	hom.	early childhood	F	n.s.
[15]	1_9del **	-	1_9del **	-	hom.	early childhood	F	yes
[16]	58G > T **	E20X	58G > T **	E20X	hom.	early childhood	M	yes
[16]	58G > T **	E20X	58G > T **	E20X	hom.	N/A	M	no
[16]	58G > T **	E20X	58G > T **	E20X	hom.	N/A	F	no
**Other MD-related plectinopathy reports**								
[67]	3064C > T	Q1022Ter	11503G > A	G3835S	c.het.	4 years	F	no
[67]	3064C > T	Q1022Ter	11503G > A	G3835S	c.het.	16 years	F	no
[68]	6118C > T	R2040W	10063T > A	F3355I	c.het.	2 years	M	yes

Ref = Reference, MD = muscular dystrophy, MB = muscle biopsy, EBS = epidermolysis bullosa simplex, dup = duplication, del = deletion, hom. = homozygous, c.het. = compound heterozygous, dev. = developed, F = female, M = male, N/A = not available, n.s. = biopsy performed, but data not shown in the respective publication, MyS = myasthenic syndrome, PA = pyloric athresia, LGMDR17 = limb girdle musculyr dystrophy type R17. Plectin mutations are listed according to the respective plectinopathy phenotype and position within the gene. Mutations are assigned to the common reference sequence, P1c (also referred to as transcript variant 1 in the databases; GenBank accession no. NM_000445). * Intronic deletion resulting in alternative splicing. ** Mutations in exon 1f, resulting in a lack of P1f and not affecting other plectin isoforms.

**Table 2:** Myopathological features of plectinopathy patients.

Ref	Mutation	Histological Analysis	Des	Ultrastructural Analysis
**EBS-MD**				
[26]	954_956dupGCT 4222C > T	irregular distribution of nuclei, variation in fiber size and shape	N/A	Z- and I-band alterations, disoriented fibers, misplaced/degenerating mitochondria, slightly altered postsynaptic cleft system
[28]	968G > A 4840G > T	cytoplasmatic nuclei, variation in fiber size, and widened interfiber spaces	+	N/A
[24]	2264_2266del 3119_3210del	myopathic pattern, fibers with rubbed-out lesions	+	degenerating myofibrils, autophagic vacuoles
[19]	2677_2685del 4930C > T	variation in fiber size, atrophic fibers, occasional de-/regenerating fibers, internal nuclei, sarcolemmal nuclear aggregates	+	subsarcolemmal clustering of mitochondria (dumbbell-shaped), paracrystalline inclusions
[24]	5018_5036del	myopathic pattern, fibers with rubbed-out lesions, COX-negative fibers	+	N/A
[48]	5728C > T	variation in fiber size, hypertrophic and atrophic fibers	+	disorganization in myofibrils and sarcomeres
[49]	5815delC	variation in fiber size, very small vacuoles at the edge of occasional fibers	N/A	loss of sarcomere organization and Z-line-plasma membrane anchorage
[49]	5854_5855del	very mild variation in fiber size	N/A	myocyte disorganization, space between the membrane and the plasma membrane is enlarged
[22]	5902_5093del 9109_9125del	fiber necrosis and regeneration, variation in fiber size, atrophic fibers, increased amounts of connective and fatty tissue	N/A	increased space between sarcolemma and sarcomere, myofibrillar disorganization, sarcomere disorganization, glycogen inclusions
[49]	7393C > T	variation in fiber size, atrophic and split fibers, central nuclei, increased amount of connective tissue	N/A	loss of normal sarcomere organization and Z-line-plasma membrane anchorage
[23,24]	13459_13474dup	myopathic pattern, rimmed vacuoles, fibers with rubbed-out lesions	+	degenerating myofibrils, cytoplasmic bodies, abnormally shaped mitochondria with paracrystalline inclusions
**EBS-MD-MyS**				
[56]	IVS11 + 2T > G 10187_10190del	variation in fiber size, mild increase in fat and connective tissue, inflammatory infiltrates, predominance of type 2 fibers	N/A	N/A
[57]	1500_1501ins36	necrotic fibers, endomysial fibrosis, variation in fiber size, splitting of hypertrophied fibers	N/A	fragmentation of endplates, postsynaptic simplification, thread-like mitochondria
[58]	2539−2A > G 11737delC	variation in fiber size, internalized myonuclei, COX-negative fibers, rubbed-out lesions, increase in connective tissue	N/A	N/A
[60,61]	6169C > T 12043dupG	variation in fiber size, necrotic and regenerating fibers, increased fibrous and fatty tissue	N/A	apoptotic nuclei, nemaline rods, disarrayed myofibrils, thick-filament loss, vacuolar change, endplate degeneration of the junctional folds
[61]	6955C > T 12043dupG	necrotic and regenerating fibers, variation in fiber size, endomysial fibrosis, clusters of large nuclei at periphery	N/A	large nuclei, clusters of mitochondria, aberrant and disrupted myofibrils, NMJ destruction of the junctional folds
**LGMDR17** (**P1f mutation**)				
[14]	1_9del *	variation in fiber size, internal nuclei, scattered basophilic and few necrotic fibers, mild endomysial fibrosis, predominance of type 2 fibers	N/A	membrane duplications, enlarged space between the membrane and sarcomere, misaligned Z-disks, glycogen inclusions
[15]	1_9del *	myopathic changes, internal nuclei, angular atrophic fibers	N/A	N/A
[16]	58G > T^*^	variation in fiber size, central nuclei, fibrosis, swollen endomysium	+	Z-line disorganization, enlarged space between the membrane and sarcomere, clusters of mitochondria
**Other MD-related plectinopathy reports**				
[68]	6118C > T 10063T > A	dystrophic features, variation in fiber size, scattered necrotic fibers and mild endomysial fibrosis	N/A	N/A

Ref = reference, Des = desmin aggregates, dup = duplication, del = deletion, N/A = not available, + = desmin aggregates reported, COX = cytochrome c oxidase, NMJ = neuromuscular junction. Plectin mutations are listed according to the respective plectinopathy phenotype and position within the gene. Mutations are assigned to the common reference sequence, P1c (also referred to as transcript variant 1 in the databases; GenBank accession no. NM_000445). * Mutations in exon 1f resulting in a lack of P1f without affecting other plectin isoforms.
